# One Health Perspective of Mycoplasmas: Proposals for Standardizing In Vitro PK/PD Models of Human Mycoplasma Species by Referencing Veterinary Experiences

**DOI:** 10.3390/microorganisms14081823

**Published:** 2026-08-18

**Authors:** Na Wang, Yang Liu, Li Xiao

**Affiliations:** 1 Department of Pulmonary and Critical Care Medicine, Huadong Hospital, Fudan University, Shanghai 200040, China; 2 Institute of Antibiotics, Huashan Hospital, Fudan University, Shanghai 200040, China; 3 Department of Medicine, University of Alabama at Birmingham, Birmingham, AL 35294, USA

**Keywords:** *Mycoplasma pneumoniae*, *Mycoplasma genitalium*, pharmacokinetics/pharmacodynamics (PK/PD), in vitro models, veterinary methodologies, One Health, antimicrobial resistance

## Abstract

The rising prevalence of macrolide and fluoroquinolone resistance in human pathogenic mycoplasmas, such as *Mycoplasma pneumoniae* and *Mycoplasma genitalium*, threatens global public health. Mutations in 23S rRNA and quinolone resistance-determining regions (QRDR) have compromised the first-line therapies, creating an urgent clinical need for alternatives. Consequently, novel antimicrobial agents (pleuromutilins, novel tetracyclines, topoisomerase inhibitors) have become a top research priority. However, standardized in vitro pharmacokinetic/pharmacodynamic (PK/PD) methodologies for mycoplasmas remain underdeveloped compared to typical bacteria. Key challenges include the lack of a cell wall, slow growth kinetics, and fastidious culture requirements, which invalidate standard colony-counting and susceptibility testing protocols. This review evaluates current in vitro PK/PD models, quantification techniques, and key indices, highlighting the potential of adapting the methodological frameworks established in veterinary mycoplasma research (e.g., *Mycoplasma bovis*, *Mycoplasma hyopneumoniae*) to human mycoplasma species. We specifically analyze the utility of propidium monoazide (PMA)-qPCR for viability assessment and hollow-fiber infection models (HFIM) configured for small-cell retention. We conclude that harmonizing these methodologies through a One Health approach is critical for optimizing dosing regimens, reducing resistance, and accelerating the development of novel antimicrobial agents for human use.

## 1. Introduction

Human pathogenic mycoplasmas, particularly *Mycoplasma pneumoniae* (*M. pneumoniae*) and *Mycoplasma genitalium* (*M. genitalium*), are significant causes of respiratory and urogenital tract infections, respectively, affecting millions of individuals worldwide [1,2]. *M. pneumoniae* is a leading cause of community-acquired pneumonia (CAP), especially in children and young adults, while *M. genitalium* is increasingly recognized as a major etiologic agent of non-gonococcal urethritis and pelvic inflammatory disease [1,3]. Historically, macrolides have been the cornerstone of treatment for mycoplasma infections, especially for pediatric populations where tetracyclines and fluoroquinolones are often restricted due to safety concerns [1]. However, recent epidemiological data showed alarming rates of resistance for both organisms, with macrolide resistance in *M. pneumoniae* exceeding 90% in some Asian regions and rising steadily in Europe and North America [1,4].

Resistance to macrolides is primarily driven by point mutations in the 23S rRNA gene at positions A2058 and A2059 (*E. coli* numbering), which prevent the drug from binding to the small subunit of bacterial ribosome [1]. Similarly, resistance to fluoroquinolones is emerging due to mutations in the quinolone resistance-determining regions (QRDR) of the genes encoding DNA gyrase and DNA topoisomerase, primarily *gyrA* and *parC* genes in mycoplasmas which affect DNA synthesis [5,6]. This resistance profile limits therapeutic options, leads to treatment failures and prolonged symptoms, and necessitates the exploration of novel antimicrobial classes with distinct mechanisms of action [7]. Several novel agents, such as lefamulin, omadacycline, and zoliflodacin, are currently in various stages of clinical development or have received recent regulatory approval [8,9,10,11,12].

To optimize their clinical use and prevent the rapid emergence of resistance to these new drugs, robust in vitro PK/PD studies are essential to provide pharmacological data for making an appropriate treatment guideline [13]. These studies help determine dosing regimens that maximize bacterial killing while minimizing the selection of resistant subpopulations within the mutant selection window (MSW). However, due to their lack of a cell wall and slow growth kinetics, mycoplasmas challenge standard PK/PD assessment models designed for conventional bacteria [14]. Standard 24 h time–kill assays are often insufficient, and the difficulty of visualizing microcolonies renders traditional colony-forming unit (CFU) counting labor-intensive and error-prone [15,16,17]. Consequently, alternative quick and accurate quantification methods need to be developed and internationally standardized. Additionally, the currently observed big variability in drug activity arises from the fastidious nature of mycoplasma culture, where media supplements, such as serum, affect drug availability via protein binding, further complicating data interpretation [15,18].

**Figure 1:**
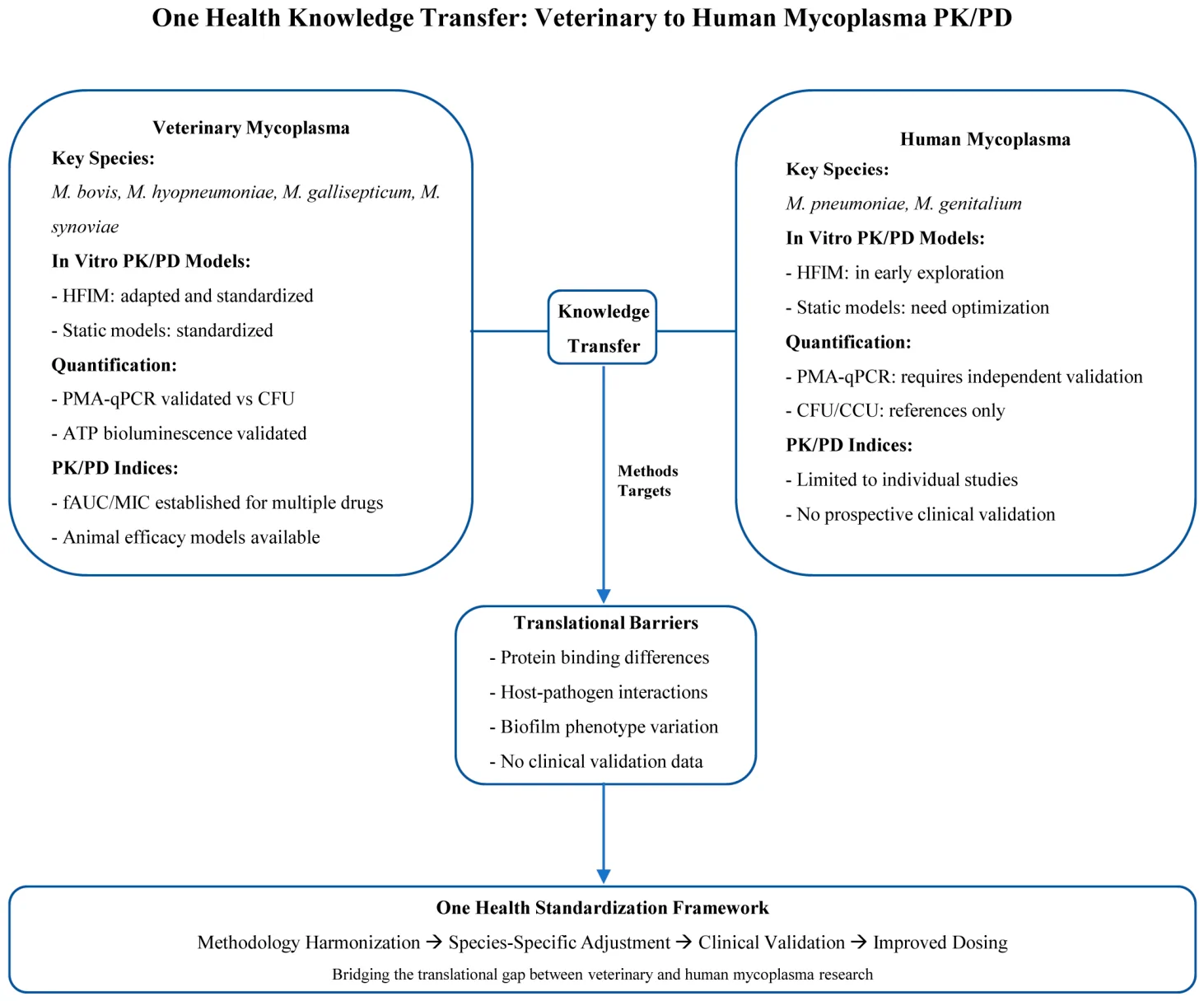
One Health knowledge transfer model comparing veterinary and human mycoplasma PK/PD methodologies, with identified translational barriers. Mature methodological frameworks established in veterinary mycoplasmas are contrasted with the current status in human pathogenic mycoplasmas, and the major translational barriers, including protein binding differences, host–pathogen interactions, biofilm phenotype variation, and the absence of clinical validation data, are summarized. A One Health standardization framework proceeding from methodology harmonization to improved dosing is proposed to bridge the translational gap.

Notably, veterinary research on economically significant mycoplasma species (e.g., *Mycoplasma bovis* in cattle and *Mycoplasma hyopneumoniae* in swine) has established more mature methodological frameworks for handling these biological constraints [19,20]. Due to the significant economic impact of veterinary mycoplasmosis on the livestock industry, substantial resources have been invested in optimizing culture conditions, dynamic models, and molecular quantification techniques for animal pathogens. This review aims to critically evaluate the current in vitro PK/PD methodologies established in animal mycoplasmas and their potential application to human pathogenic mycoplasmas, as well as to identify gaps and limitations in current human-focused studies. Special emphasis is placed on discussing the strategies of adapting veterinary-derived techniques for quantification (e.g., propidium monoazide (PMA)-qPCR) and dynamic modeling (e.g., hollow-fiber infection model (HFIM) configurations) to bridge the current methodological gap in studies for human species. By integrating these cross-species insights, we propose a roadmap for standardizing mycoplasma PK/PD research under a ‘One Health’ framework, which will ultimately improve the predictability of clinical outcomes. The comparison of major methods and their potential transfer to human application under a One Health perspective are summarized in Table 1 and Figure 1.

We performed a literature search of PubMed, Web of Science, and Scopus databases (up to June 2026) using combinations of the following keywords: “Mycoplasma”, “mycoplasma”, “PK/PD”, “pharmacokinetics”, “pharmacodynamics”, “in vitro models”, “antimicrobial susceptibility”, “veterinary”, and “One Health”. We included original research articles, reviews, and guideline documents that addressed in vitro PK/PD methodology for human or veterinary mycoplasma species. Reference lists of included articles were screened for additional relevant studies. A total of 71 references were ultimately included.

## 2. Biological Barriers to Standard PK/PD Methodologies

The successful implementation of PK/PD studies relies on the assumption that the in vitro model accurately reflects the biological behavior of the pathogen under drug pressure. However, mycoplasmas present unique biological barriers that invalidate many standard protocols used for other bacteria. Understanding these barriers is the first step to adapting methodologies from other fields, including veterinary science.

### 2.1. Lack of Cell Wall and Formation of Cell Aggregates

A defining characteristic of the class Mollicutes is the complete absence of a peptidoglycan cell wall, distinguishing it from most other bacteria [1]. While the fundamental principle of a viability assessment remains the capacity to form colonies on solid media—the gold standard for typical bacteria like *E. coli* or *S. aureus*—this structural difference, combined with slow growth kinetics and colony size, renders standard CFU enumeration impractical for routine use [1,29]. In conventional bacteriology, bactericidal activity is quantified by the inability to form colonies, a process often facilitated by antibiotic-induced osmotic lysis (e.g., beta-lactams) that enables rapid discrimination between viable and non-viable cells. For mycoplasmas, however, defining ‘bactericidal’ activity is markedly more challenging because the absence of a cell wall prevents osmotic lysis; cells may retain morphological integrity and membrane continuity despite an irreversible loss of reproductive capacity [30]. Consequently, confirming bactericidal effects cannot rely on rapid morphological cues and instead depends on a prolonged culture-based enumeration of microcolonies (typically 3–7 days) or alternative endpoints such as metabolic activity (e.g., color-changing units, CCU), viability-associated genomic quantification, or membrane integrity staining [1]. This methodological dependency introduces susceptibility to confounding factors, including residual drug carryover, media variability, and subjective interpretation of microcolony boundaries, thereby increasing inter-laboratory variability [22,26]. Ultimately, this necessitates a shift from CFU/mL to CCU/mL or adjusted genomic metrics in PK/PD calculations, complicating data comparison with standard bacteria and requiring specific validation of what constitutes a ‘log kill’ in the absence of cell lysis [31].

In addition to the lack of cell walls, mycoplasmas express adhesins on the cell surface and form cell aggregates in culture suspension. A study on *M. pneumoniae* showed that different methods for the disruption of the aggregates cause high variations in CFU enumeration and colony phenotypes and a brief and gentle sonication generates a 10-fold enrichment of recoverable CFU compared to other methods [32]. Notably, sonication parameters require species- and strain-specific optimizations; excessive sonication may damage cells and bias viability estimates.

### 2.2. Slow Growth Rate

Human pathogenic mycoplasmas exhibit generation times ranging from 6 to 20 h, significantly slower than *S. aureus* (approx. 30 min) or *E. coli* (approx. 20 min) [33]. For *M. pneumoniae* or *M. genitalium*, the growth rate is even slower, often requiring 1–6 weeks for visible culture growth, posing extreme challenges for PK/PD timelines [33]. Standard 24 h time–kill assays are insufficient to capture full growth kinetics and drug effects, often leading to premature conclusions of efficacy or failure to detect delayed killing [15]. PK/PD studies on veterinary mycoplasma species often extend observation periods to 72 h or longer to ensure accurate endpoint determination and capture delayed killing effects [19,23]. Using a 24 h window may lead to inaccurate PK/PD target calculations, potentially underestimating the required drug exposure for eradication. Therefore, studies on human mycoplasma species may benefit from referencing these extended incubation windows, although direct validation in human mycoplasma PK/PD studies is still needed [14]. Mathematical models used to fit kill curves must also account for this lag phase to avoid skewing the *E*_max_ parameters.

### 2.3. Fastidious Culture Requirements

Mycoplasma cultivation requires complex media supplemented with sterols (e.g., cholesterol), serum, yeast extract, and specific pH indicators like phenol red [27]. Common media include SP4, Hayflick, or Friis broth, each with varying protein and lipid contents that can influence drug activity. High protein content in media can bind antimicrobial agents (e.g., macrolides, tetracyclines), altering the free drug concentration available for activity [1,15,19]. PK/PD indices must be calculated using free drug concentrations. This requires correction for media-specific protein binding, which differs from that of human serum [18]. Furthermore, media components like cholesterol can interact with certain drugs, potentially reducing their apparent potency in vitro [15]. Standardizing media composition across laboratories is therefore critical to ensure reproducible MIC and PK/PD target values.

### 2.4. Intracellular and Biofilm Niches

Species like *M. genitalium* and *M. pneumoniae* can invade and persist within host epithelial cells, creating a sanctuary site protected from extracellular drugs [27]. Additionally, biofilm formation has been documented in both human and veterinary mycoplasmas, contributing to increased tolerance and chronic infection [34]. Standard planktonic PK/PD models fail to account for the reduced penetration of antibiotics into host cells or biofilm matrices [35]. Therefore, in vitro models must account for intracellular penetration (measured via accumulation ratios) and biofilm penetration, not just extracellular activity. Veterinary studies on *M. bovis* biofilms have established protocols for quantifying biofilm-associated PK/PD that could be adapted for human mycoplasma [36,37]. Ignoring these niches in PK/PD modeling may lead to clinical dosing regimens that fail to eradicate persistent infections, contributing to relapse rates observed in clinical practice. However, the mechanistic basis of these intracellular and biofilm niches in mycoplasmas remains under-characterized compared with typical bacteria. Consequently, current PK/PD models for these niches are largely extrapolated from other pathogens, which represents a key evidence gap.

## 3. PK/PD Indices: Calculation and Adaptation

Determining the correct PK/PD index is fundamental for translating in vitro data into clinical dosing regimens. While the fundamental principles of PK/PD apply across various bacterial species, the specific calculation parameters require adaptation for mycoplasmas.

### 3.1. Primary Indices

The primary PK/PD indices driving efficacy include fAUC/MIC (free drug area under the concentration–time curve to MIC ratio), f*C*_max_/MIC (free peak concentration to MIC ratio), and %f*T* > MIC (percentage of free drug concertation time above MIC) [38]. For most novel agents used against mycoplasmas, particularly protein synthesis inhibitors like lefamulin and tetracyclines, fAUC/MIC has been identified as the primary predictor of efficacy [39,40]. However, for fluoroquinolones, both AUC/MIC and *C*_max_/MIC may play roles, depending on the specific compound and strain susceptibility [41]. Determining the dominant index requires rigorous regression analysis of kill kinetics against varying PK profiles, which is rarely done consistently for mycoplasmas due to methodological difficulties. To date, published human mycoplasma PK/PD investigations remain limited. For nemonoxacin, Wang et al. established fAUC/MIC as the best predictors of efficacy against *M. pneumoniae* using an *E*_max_ model, providing a rare human-derived PK/PD dataset for this pathogen [14]. For lefamulin, Bhavnani et al. performed PK/PD target attainment analyses supporting intravenous and oral dose selections for community-acquired bacterial pneumonia, demonstrating the translational pathway from preclinical models to human dosing—although these analyses were not mycoplasma-specific [39]. Omadacycline has demonstrated favorable in vitro activity against human mycoplasmas, but its mycoplasma-specific PK/PD targets have yet to be established through dedicated dose–response studies [11]. Despite these efforts, human mycoplasma-specific PK/PD data remain scarce. The scarcity of such data underscores the importance of adapting established veterinary PK/PD frameworks while actively pursuing clinical validation in human populations.

### 3.2. Time-Window Adjustment

Calculating these indices requires defining the appropriate effect observation window. As discussed in Section 2.2, growth inhibition may not be evident until 48–72 h [15]. Mathematical modeling (e.g., *E*_max_ models) should be adjusted to account for this lag phase in growth inhibition [14]. A study on *M. hyopneumoniae* suggests normalizing growth rates before calculating PK/PD targets to account for species-specific generation times [24]. For example, a 1-log reduction in mycoplasma load may take 48 h, whereas in *S. aureus* it may take 8 h; thus, the rate of kill (slope) is a critical parameter to report alongside the target index [25].

### 3.3. Mutant Prevention Concentration (MPC) and Inoculum Effect

The MPC is crucial for suppressing resistance emergence by blocking the selection of first-step mutants [42]. Determining MPC for mycoplasma requires high inoculum densities (~10^10^ CCU/mL), which are difficult to achieve due to low maximum culture densities [14,43]. The Inoculum Effect, where MIC increases with higher bacterial density, is also pronounced in mycoplasmas and must be characterized for accurate PK/PD modeling [19]. Failure to account for the inoculum effect may result in underdosing in high-burden infections such as severe pneumonia. Veterinary studies have demonstrated that MPC values can vary significantly depending on the media used, reinforcing the need for standardized conditions [28].

## 4. Evolution of In Vitro PK/PD Models

**Figure 2:**
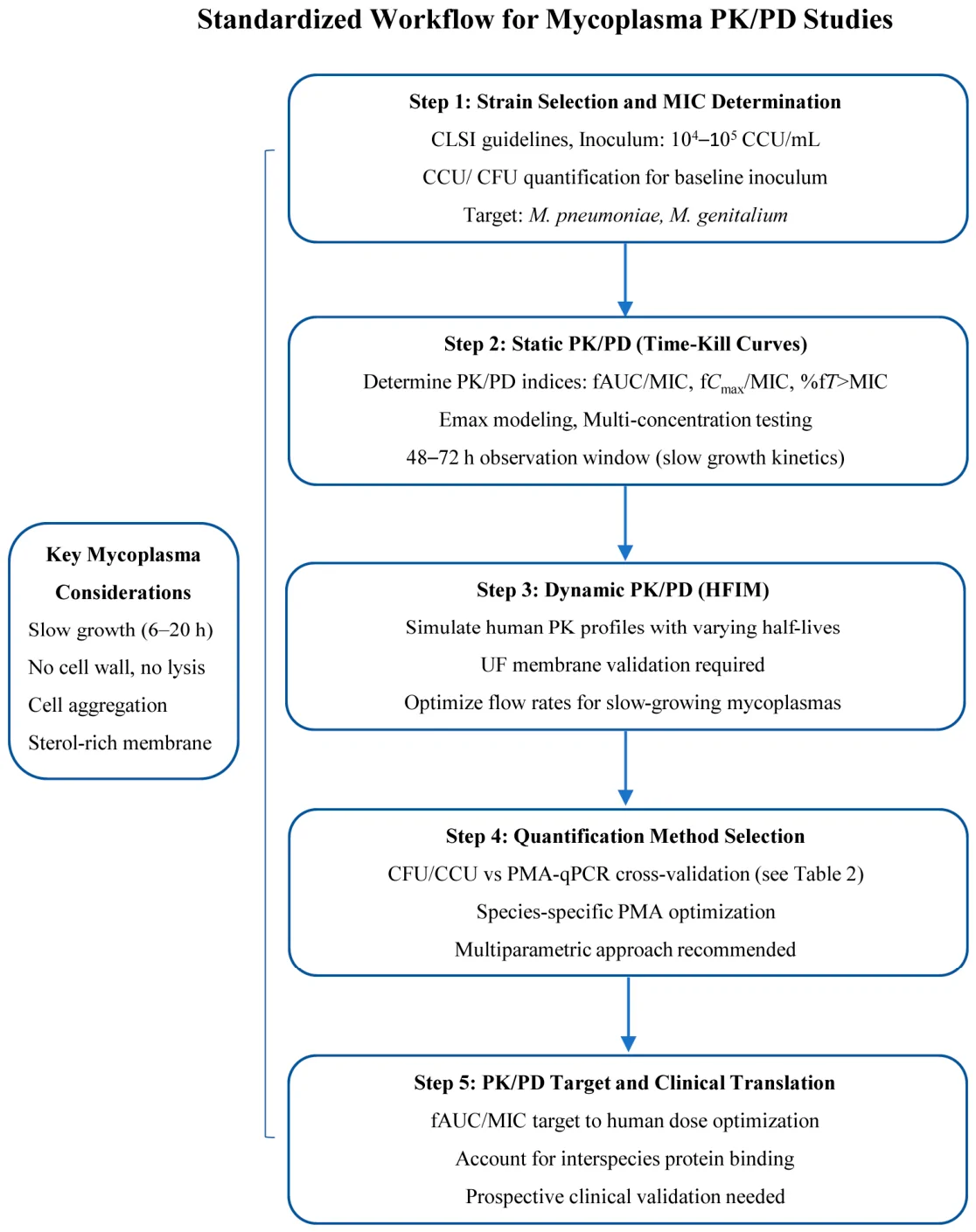
Standardized workflow for mycoplasma PK/PD studies, encompassing strain selection, static and dynamic PK/PD modeling, quantification method selection, and clinical translation. Proposed standardized workflow for mycoplasma PK/PD studies, ranging from strain selection to clinical translation. The workflow comprises five sequential steps: (1) strain selection and MIC determination, (2) static PK/PD modeling with time–kill curves, (3) dynamic PK/PD modeling using HFIM, (4) quantification method selection, and (5) PK/PD target derivation and clinical translation, with prospective clinical validation identified as the final requirement.

The choice of in vitro model dictates the quality of PK/PD data. For mycoplasma, models must be adapted to accommodate their unique biological characters. An overview of the standardized workflow from strain selection to clinical translation is presented in Figure 2.

### 4.1. Static Models

Static models, such as broth microdilution, are the most common method for MIC determination and basic time–kill studies [15]. However, a visual reading of color change (phenol red) is subjective, low-throughput, and prone to inter-observer variability [15]. Veterinary studies have implemented automated spectrophotometric reading to objectify color change and improve reproducibility [21]. Additionally, the use of metabolic indicators like Alamar Blue (resazurin) has been validated in veterinary mycoplasma studies for faster readouts [17]. Adopting these standardized reading criteria and metabolic indicators could reduce variability in human PK/PD studies, though this approach requires further validation in human mycoplasma species. Standardization of static models is the first step towards more complex dynamic modeling. Furthermore, static models fail to simulate the fluctuating drug concentrations seen in patients, limiting their predictive value for dosing intervals [14,44,45].

### 4.2. Dynamic Models: Hollow-Fiber Infection Model (HFIM)

The HFIM simulates human pharmacokinetic profiles dynamically, allowing for varying half-lives and dosing intervals [46]. It is considered the gold standard for PK/PD modeling in bacteria. However, its application to mycoplasmas faces unresolved challenges, including membrane retention and washout of slow-growing organisms, and the trade-offs between different membrane configurations warrant critical evaluation. For mycoplasma-related experiments, conventional microporous fiber membranes with pore sizes of 0.1 μm and 0.22 μm are generally unsuitable. Due to their highly pleomorphic structure, mycoplasmas can readily deform and penetrate these membrane pores, which will cause obvious biomass loss [27]. Although ultrafiltration (UF) membranes can provide a superior interception performance, there is no universally recognized standard for their pore size classification; UF membranes are merely defined by molecular-weight cutoff [47]. As a result, retention efficiency and drug permeability vary among products from different manufacturers. Comprehensive experimental verification is urgently needed before UF membranes can be reliably applied to mycoplasma HFIM studies. We propose that each membrane lot be pre-tested for mycoplasma retention and drug diffusion, with acceptance criteria defined based on the specific experimental objectives and membrane type [47]. Furthermore, high media flow rates may wash out slow-growing organisms before they can replicate; therefore, optimized flow rates (e.g., lower dilution rates) should be considered to maintain stable mycoplasma populations. System volume and sampling frequency may also require adjustments to account for the slower kinetics of mycoplasma killing. Despite these challenges, HFIM remains the most promising tool for simulating human dosing regimens for mycoplasma [47].

### 4.3. Biofilm Models

Biofilm-associated infections are increasingly recognized in mycoplasmosis, and biofilm-associated antibiotic resistance have been observed in *M. genitalium* by in vitro studies [48]. Static microtiter plate assays are commonly used to induce biofilm formation over 3–7 days [48]. Quantification often involves Crystal Violet staining for biomass or XTT assays for metabolic activity within the biofilm [34]. PK/PD studies within these biofilm models are needed to determine penetration efficacy and biofilm-specific MICs (MBIC). In other bacterial species, biofilm MICs have been reported to be substantially higher than planktonic MICs, with some studies showing up to 10–100-fold increases [49]. However, whether such a magnitude of increase directly applies to mycoplasma biofilms remains to be determined, as mycoplasma-specific biofilm PK/PD data remain limited [48,50]. Incorporating biofilm models into early drug development could prevent clinical failures in chronic infections [51]. However, standardizing biofilm induction methods remains a challenge across laboratories [50].

## 5. Advances in Quantification Techniques

Accurate quantification of bacterial load is the cornerstone of PK/PD analysis. For mycoplasmas, this remains one of the most significant methodological challenges due to slow growth kinetics, lack of turbidity, and microscopic colony size [27].

### 5.1. Conventional Culture-Based Methods

CCU and CFU are the conventional standards for mycoplasma quantification [15,29,52]. CCU relies on metabolic acid/base production altering a pH indicator (e.g., phenol red), with the endpoint defined as the highest dilution showing color change [52]. While standardized for routine MIC testing (IRPCM recommends 10^3^–10^5^ CCU/mL; CLSI recommends 10^4^–10^5^ CFU/mL), CCU is inherently indirect and time-consuming (typically 3–14 days) [52]. It is also subject to personalinterpretation, particularly near the threshold of detection [15]. Veterinary protocols have attempted to improve objectivity by adopting spectrophotometric readings (e.g., OD550) to quantify color shift, enabling higher throughput [21]. However, for PK/PD time–kill studies requiring frequent, precise viability measurements, conventional culture methods are often too slow and lack the temporal resolution needed to capture dynamic killing kinetics. CFU enumeration via solid agar provides a more direct measure of reproductive viability but is labor-intensive, requires stereomicroscopy, and shares the same temporal limitations [14]. Moreover, mycoplasma colonies are exceptionally small (typically 10–100 μm in diameter) and often grow embedded at different depths within the agar matrix rather than solely on the surface. This three-dimensional distribution necessitates focusing through multiple planes under stereomicroscopy, significantly increasing counting time, operator fatigue, and inter-observer variability [53]. Colonies at different depths may also exhibit differential growth rates and antibiotic exposure, further complicating accurate enumeration [53].

### 5.2. qPCR Limitations in Viability Assessment

Quantitative PCR (qPCR) offers high sensitivity and rapid turnaround, with common targets including 16S rRNA gene, *gap*, or *parC* genes depending on the species [54]. However, standard qPCR amplifies DNA from both viable and non-viable cells, which can significantly overestimate bacterial load in PK/PD studies where killing efficiency is the primary endpoint [55]. This limitation may mask true bactericidal activity and distort kill-curve modeling. Furthermore, extracellular DNA from lysed cells can persist in culture media for hours to days, further confounding viability estimates [56]. Consequently, while qPCR is highly suitable for MIC determination (growth vs. no-growth thresholds), it requires modification to accurately reflect viable cell dynamics in time–kill assays.

### 5.3. Propidium Monoazide (PMA)-qPCR: Opportunities and Limitations

PMA-qPCR offers a promising approach to distinguish viable from non-viable cells in conventional bacterial systems [57]. PMA selectively penetrates membranes of dead or compromised cells, intercalates into DNA, and prevents amplification upon photoactivation [57]. This technique has been extensively validated for other common bacterial pathogens but remains largely unexplored in mycoplasma research, with validation to date limited to *M. hyopneumoniae* and other veterinary species [26] and only scant data available for human mycoplasmas [1,58,59]. The veterinary findings about PMA-qPCR cannot be directly extrapolated to human mycoplasma species without species-specific validation. Given the unique membrane composition of mycoplasmas (sterol-rich, cell wall-deficient), direct translation of PMA-qPCR protocols from conventional bacteria requires careful optimization and validation [27]. Key parameters including PMA concentration, light exposure duration, and membrane permeability thresholds may differ substantially due to mycoplasma-specific characteristics [26,60]. Moreover, incomplete exclusion of DNA from dead cells can overestimate viability [55]. Potential misclassification of injured but viable cells could further confound viability estimate [60]. These considerations underscore that PMA-qPCR, while promising, remains an emerging methodology requiring substantial species-specific optimization rather than a validated replacement for culture-based endpoints. Future methodological development should prioritize: (1) establishing PMA penetration kinetics in mycoplasma membranes; (2) validating against gold-standard CCU/CFU endpoints; and (3) optimizing for species-specific variations in membrane composition [15]. Coupling this approach with internal amplification controls would further normalize for extraction efficiency and PCR inhibition, potentially providing a reliable, high-throughput metric for kill kinetics once validated.

### 5.4. Other Techniques

Alternative quantification methods offer distinct advantages but require species-specific optimization. Flow cytometry with viability dyes (e.g., SYTO 9/propidium iodide) enables single-cell resolution and rapid enumeration; however, the small size of mycoplasma (~0.2–0.3 μm) necessitates high-sensitivity instruments and rigorous gating to exclude media debris [61,62]. ATP bioluminescence provides a rapid metabolic readout but is frequently compromised by background interference from serum-rich media [63]. Digital PCR (dPCR) delivers absolute quantification without standard curves but, like conventional qPCR, cannot differentiate live from dead cells without prior viability treatment [64]. Furthermore, its routine implementation is often constrained by high operational costs and limited instrument accessibility compared to standard qPCR platforms [65]. Given the trade-offs of each method, as compared in Table 2, a multiparametric approach—such as using PMA-qPCR for high-frequency time–kill sampling validated against periodic CCU/CFU endpoints—currently represents a promising and practical strategy.

### 5.5. Translational Limitations and Clinical Relevance

Although veterinary PK/PD models offer valuable methodological insights for human mycoplasma research, several translational limitations must be acknowledged [66,67]. First, host–pathogen interactions differ substantially between veterinary and human hosts; infection sites (e.g., bovine respiratory tract vs. human lung), immune responses, and disease progression kinetics may not be directly comparable [68]. Second, the inoculum burden and biofilm phenotypes observed in veterinary infections may not reflect those in human *M. pneumoniae* or *M. genitalium* infections, potentially affecting the clinical relevance of PK/PD targets derived from animal models [69]. Third, protein binding profiles vary significantly across species—veterinary PK/PD targets based on free drug concentrations in bovine or swine serum may not translate directly to human serum binding [18]. Fourth, the available clinical data linking in vitro PK/PD targets to human treatment outcomes for mycoplasma infections remain extremely limited; most existing correlations are derived from other bacterial infections (e.g., *S. pneumoniae*) rather than mycoplasma-specific studies [70]. Fifth, differences in drug pharmacokinetics between animal models and humans further complicate the direct translation of veterinary PK/PD targets. Sixth, mycoplasma species differ substantially in their biology (e.g., tissue tropism, growth requirements, and pathogenicity mechanisms); a PK/PD target established for *M. bovis* in cattle may not be directly applicable to *M. pneumoniae* or *M. genitalium* in humans, even if the in vitro methodology is transferable. Seventh, the lack of standardized clinical outcome definitions for mycoplasma infections hampers the validation of in vitro targets against patient outcomes. Finally, regulatory considerations, including the absence of harmonized breakpoint-setting frameworks for mycoplasma across human and veterinary medicine, represent an additional barrier to the implementation of a One Health approach. These limitations underscore that veterinary methodologies should be regarded as valuable starting points requiring systematic adaptation and validation for human applications.

It is important to note that many of the methodological recommendations discussed in this review are based on limited or preliminary evidence, often derived from single studies or single veterinary species. Conflicting findings exist in the literature; for example, reported optimal PMA concentrations and light-exposure durations vary considerably across studies, and the transferability of these parameters to human mycoplasmas—which differ fundamentally from those of typical bacteria—remains uncertain. Similarly, the relative performance of CCU versus CFU as endpoints has been debated, with each method carrying distinct biases that may affect PK/PD target estimation. Acknowledging these methodological uncertainties is essential, as premature standardization of inadequately validated protocols could introduce systematic errors into PK/PD data. Future research should prioritize prospective clinical validation of these in vitro targets in human populations to close the in vitro–in vivo correlation gap. Until such data are available, animal-derived PK/PD benchmarks should be interpreted cautiously and used primarily as guiding references rather than definitive clinical dosing standards.

Regarding the practical implementation of this One Health approach, we acknowledge that its realization depends on several prerequisites. The likelihood of implementation would be enhanced by establishing cross-disciplinary platforms connecting veterinary and human medicine, such as joint antimicrobial susceptibility surveillance programs and shared reference laboratories for mycoplasma testing. Potential obstacles include the current separation of veterinary and human regulatory frameworks, limited funding for comparative mycoplasma research, and the scarcity of mycoplasma-focused PK/PD expertise in both fields. Professional societies, public health agencies, and academic consortia specializing in antimicrobial resistance could take the initiative to coordinate such collaborative efforts, supported by national and international funding programs.

## 6. Conclusions

Robust in vitro PK/PD methodologies are essential for combating resistant mycoplasma infections and optimizing novel antimicrobial use. This review highlights that veterinary research offers valuable, validated technical solutions for quantification (PMA-qPCR) and modeling (HFIM configurations). Key biological barriers such as slow growth and lack of a cell wall require specific adaptations. Research on human mycoplasma species should consider referencing veterinary protocols, recognizing that species-specific validation remains necessary. Standardizing these methods will accelerate the development and optimal use of novel antimicrobial agents, ultimately improving patient outcomes. Future research should focus on validating these in vitro targets against clinical data to close the in vitro–in vivo correlation gap. A collaborative ‘One Health’ effort is the most efficient path forward for mycoplasma PK/PD standardization. By leveraging methodological advancements from veterinary science, human medicine can overcome the current technical bottlenecks and better address the growing threat of resistant mycoplasma infections. However, these approaches should currently be regarded as promising frameworks rather than established standards. Species-specific validation, standardization of protocols, and, critically, prospective clinical validation of in vitro targets against patient outcomes are required before these methodologies can be reliably applied to guide clinical dosing. A collaborative ‘One Health’ effort offers the most efficient path forward, provided that these evidence gaps are systematically addressed.

## Figures and Tables

**Table 1:** Comparison of veterinary and human mycoplasma PK/PD methodological frameworks from a One Health perspective.

Aspect	Veterinary	Human	Cross-Applicability	Level of Evidence *
Key species	*M. bovis*, *M. hyopneumoniae*, *M. gallisepticum* [21,22,23]	*M. pneumoniae*, *M. genitalium* [1,2]	Biologically similar; methods potentially transferable	Validated
In vitro PK/PD models	HFIM successfully adapted; static models standardized [24,25]	HFIM in early exploration; static models need optimization [14]	HFIM protocols adaptable with membrane validation	Partially Validated
Quantification	PMA-qPCR validated against CFU [22,26]	PMA-qPCR requires independent validation [15,27]	Protocol transfer possible with species-specific optimization	Partially Validated
PK/PD indices	fAUC/MIC established for multiple drugs [23,24,25]	Limited to individual studies [14] (e.g., nemonoxacin)	Conceptual framework transferable; targets need recalibration	Partially Validated
Clinical validation	Animal efficacy models available [23,28]	Lack of prospective clinical validation (see Section 5.5)	Translational limitations must be addressed	Proposed

* Level of Evidence: Validated, supported by multiple independent studies; Partially Validated, supported by preliminary or single-study evidence, mainly in veterinary species, requiring confirmation in human mycoplasmas; Proposed, based on extrapolation or theoretical rationale without direct validation.

**Table 2:** Comparison of quantification methods for mycoplasma PK/PD studies.

Method	Principle	Throughput	Turnaround Time	Mycoplasma-Specific Challenges	Recommended Application
CFU	Colony counting on solid agar	Low	3–14 days	Microscopic colonies embedded in agar matrix; stereomicroscopy required; prone to aggregation [53]	Gold standard for PK/PD endpoint verification
CCU	Metabolic acid production alters pH indicator (phenol red)	Medium	3–14 days	Subjective endpoint interpretation; low temporal resolution for kill kinetics [15,52]	Routine MIC testing; growth/no-growth threshold assays
qPCR	DNA amplification	High	2–4 h	Amplifies DNA from both viable and non-viable cells; extracellular DNA persistence in culture media [54,55,56]	Rapid monitoring; not suitable as PK/PD sole endpoint
PMA-qPCR	Selective amplification of viable-cell DNA after membrane-impermeant dye treatment	High	3–5 h	Unique sterol-rich, wall-deficient membrane affects PMA penetration; incomplete dead-cell DNA exclusion [26,55,60]	Emerging tool requiring species-specific validation
ATP bioluminescence	Quantification of metabolic ATP as proxy for viable biomass	High	30 min– 1 h	Background interference from serum-rich media; correlation with CFU/CCU not consistently established for mycoplasma [63]	Rapid preliminary screening
Flow cytometry	Single-cell analysis using viability dyes (e.g., SYTO 9/PI)	Medium– High	1–2 h	Small cell size requires high-sensitivity instruments; rigorous gating needed to exclude debris [61,62]	Research tool for dynamic single-cell analysis

## Data Availability

The original contributions presented in this study are included in the article. Further inquiries can be directed to the corresponding authors.
